# Application of probabilistic models for extreme values to the COVID-2019 epidemic daily dataset

**DOI:** 10.1016/j.dib.2021.107783

**Published:** 2022-01-01

**Authors:** Daniel Canton Enriquez, Jose A. Niembro-Ceceña, Martin Muñoz Mandujano, Daniel Alarcon, Jorge Arcadia Guerrero, Ivan Gonzalez Garcia, Agueda Areli Montes Gutierrez, Alfonso Gutierrez-Lopez

**Affiliations:** a Facultad de Informatica, Universidad Autonoma de Queretaro, Juriquilla, Queretaro 76230, Mexico; b Facultad de Ingeniería, Universidad Autonoma de Queretaro, Centro Universitario, Queretaro 76010, Mexico; c Water Research Center, Centro de Investigaciones del Agua-Queretaro (CIAQ), International Flood Initiative, Latin-American and the Caribbean Region (IFI-LAC), Intergovernmental Hydrological Programme (IHP-UNESCO), Universidad Autonoma de Queretaro, Queretaro 76010, Mexico

**Keywords:** Daily new cases statistical analysis, Coronavirus, Gumbel distribution, Exponential distribution, Probabilistic analysis

## Abstract

Worldwide, COVID-19 coronavirus disease is spreading rapidly in a second and third wave of infections. In this context of increasing infections, it is critical to know the probability of a specific number of cases being reported. We collated data on new daily confirmed cases of COVID-19 breakouts in: Argentina, Brazil, China, Colombia, France, Germany, India, Indonesia, Iran, Italy, Mexico, Poland, Russia, Spain, U.K., and the United States, from the 20th of January, 2020 to 28th of August 2021. A selected sample of almost ten thousand data is used to validate the proposed models. Generalized Extreme-Value Distribution Type 1-Gumbel and Exponential (1, 2 parameters) models were introduced to analyze the probability of new daily confirmed cases. The data presented in this document for each country provide the daily probability of rate incidence. In addition, the frequencies of historical events expressed as a return period in days of the complete data set is provided.

**Specifications Table**

**Table:** 

Subject	Data Mining and Statistical Analysis. Infectious Diseases
Specific subject area	Generalized Extreme-Value Distribution Type 1-Gumbel and Exponential (1, 2 parameters models applied to characterize probabilistically COVID-19 daily cases
Type of data	Table Graph Figure
How the data were acquired	The data on daily recent confirmed cases of COVID-19 were carefully collected from Dashboard by the Center for Systems Science and Engineering (CSSE) at Johns Hopkins University (JHU) Database. The data were built as a time-series database by Excel and probabilistic models for extreme values were satisfactorily established for analysis using Matlab.
Data format	Analyzed
Parameters for data collection	Under the framework of frequency analysis and the Moments estimation parameter method, a probabilistic fitting was carried out to the daily new confirmed Covid cases. Raw data from Argentina, Brazil, China, Colombia, France, Germany, India, Indonesia, Iran, Italy, Mexico, Poland, Russia, Spain, U.K., and the United States, were used.
Description of data collection	Daily data on new confirmed cases of COVID-19 outbreaks in Argentina, Brazil, China, Colombia, France, Germany, India, Indonesia, Iran, Italy, Mexico, Poland, Russia, Spain, U.K., and the United States from the 20th of January, 2020 to 28th of August 2021 are available in the Database. COVID-19 Dashboard by the Center for Systems Science and Engineering (CSSE) at Johns Hopkins University (JHU) (https://coronavirus.jhu.edu/). In addition, there are no missing values and the Excel file of the daily data is presented in Supplementary Data. This is the data repository for the 2019 Novel Coronavirus Visual Dashboard operated by the Johns Hopkins University Center for Systems Science and Engineering (JHU CSSE). Also, Supported by ESRI Living Atlas Team and the Johns Hopkins University Applied Physics Lab (JHU APL) [1]. https://coronavirus.jhu.edu/map.html https://github.com/CSSEGISandData/COVID-19/blob/master/README.md
Data source location	Argentina, Brazil, China, Colombia, France, Germany, India, Indonesia, Iran, Italy, Mexico, Poland, Russia, Spain, U.K., and the United States.
Data accessibility	The analyzed data is publicly hosted in the mendeley repositories with the following data: Repository name: Frequency analysis of new Covid-19 infections Matlab code: https://github.com/dCantonE/FrequencyAnalysis Supplementary material associated with this article: https://data.mendeley.com/datasets/kvnsn8nyhg/3

**Value of the Data**

• Data on daily Covid cases are now easy to obtain. Authorities there are beginning to compile, cross-check and release these data to examine and analysis it. Thus, they are widely available in most countries. However, it is not easy to associate a probability of event occurrence to each daily case report data.
• These data can be updated through official reports and specialized websites. The database presented here is easy to update during the progress of the epidemic (including the third wave in some countries). In data-set of new daily cases are associated with their probability of frequency. They can be wielded to determine the probability of recent infections at specific sites.
• The likelihood of a new outbreak of Covid in any of the countries above can be estimated employing the extreme values probability distribution with the best fit.
• This dataset also supports expanding understanding of the differences in geographic scale in forecasting COVID-19 case counts [2]. Show that statistically significant differences exist based on percentage error metrics when using the same forecasting method at different levels of geographic resolution.
• The probability distributions presented are a complement to a forecasting model. This dataset provides daily probability of rate incidence that could be explored alongside forecasting data to gain further insight into the validity of different forecasts at varied geographic scales as a result of population size differences across countries.
• In order to provide health institutions, research centers and authorities with probabilistic tools to respond to changes in the epidemic. The Matlab code for the systematic of the frequency calculations is included.

## 1 Data Description

**Fig. 1:**
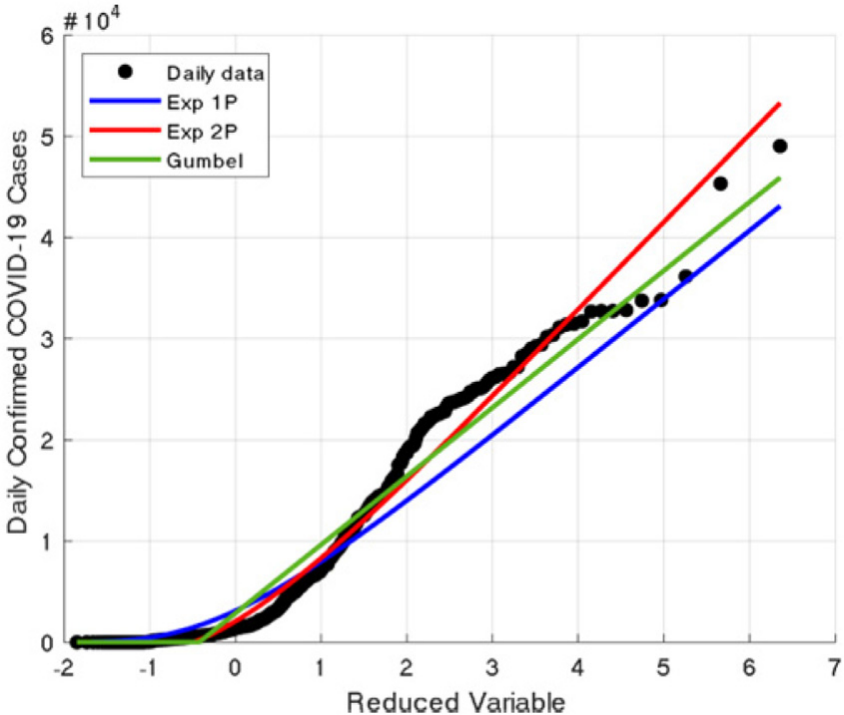
Comparison between fit proposed models Exp-1P, Exp-2P and Gumbel with daily data on confirmed cases of COVID-19 in Germany.

**Fig. 2:**
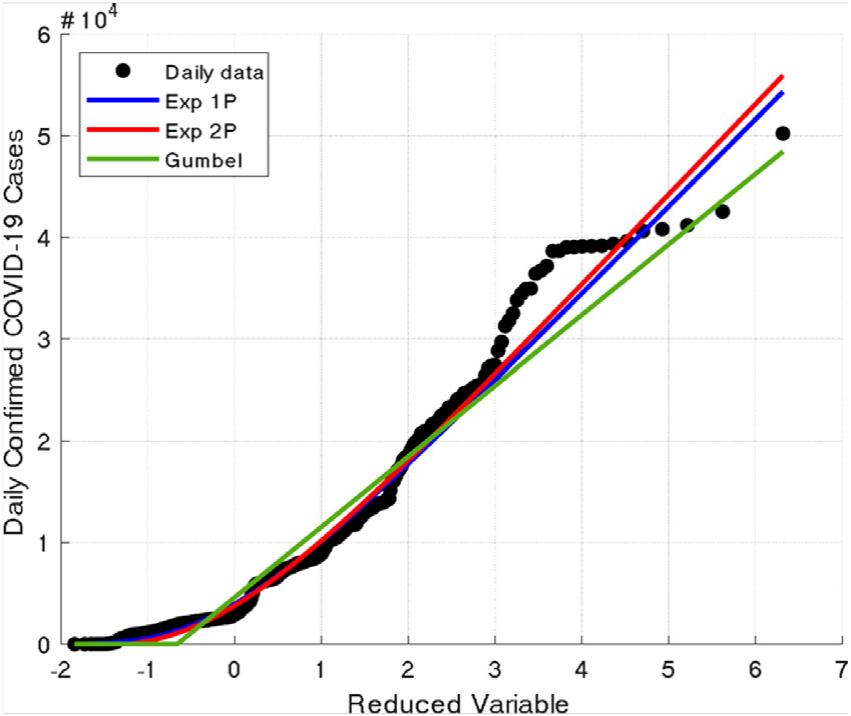
Comparison between fit proposed models Exp-1P, Exp-2P and Gumbel with daily data on confirmed cases of COVID-19 in Iran.

**Fig. 3:**
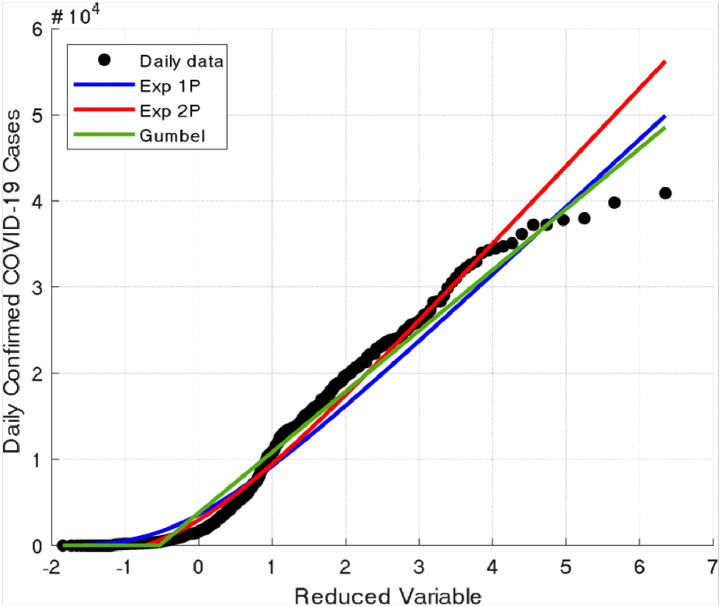
Comparison between fit proposed models Exp-1P, Exp-2P and Gumbel with daily data on confirmed cases of COVID-19 in Italy.

**Fig. 4:**
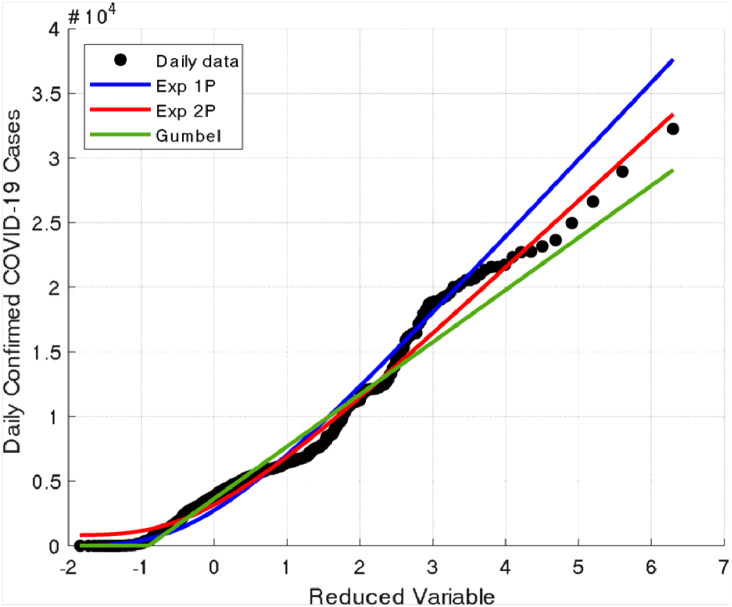
Comparison between fit proposed models Exp-1P, Exp-2P and Gumbel with daily data on confirmed cases of COVID-19 in Mexico.

**Fig. 5:**
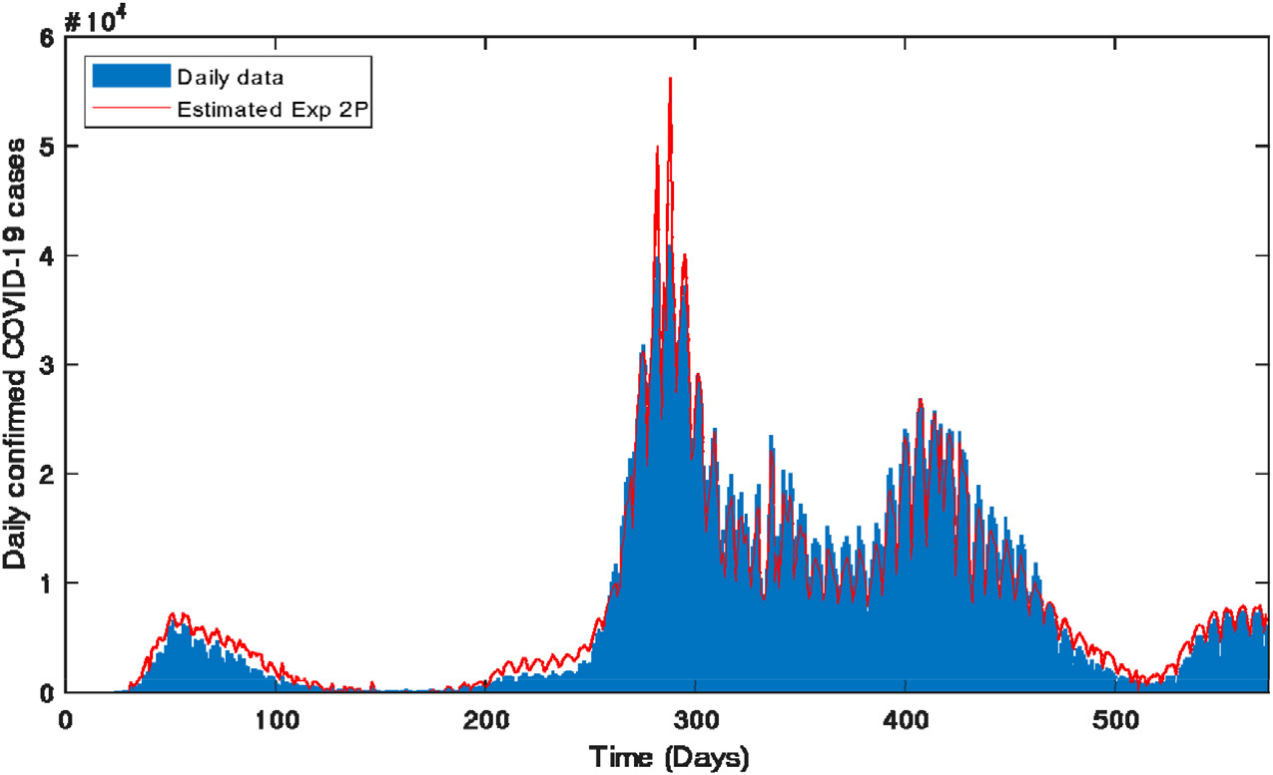
Daily new confirmed cases in Italy, probabilistic characterization with Exp-2P.

**Fig. 6:**
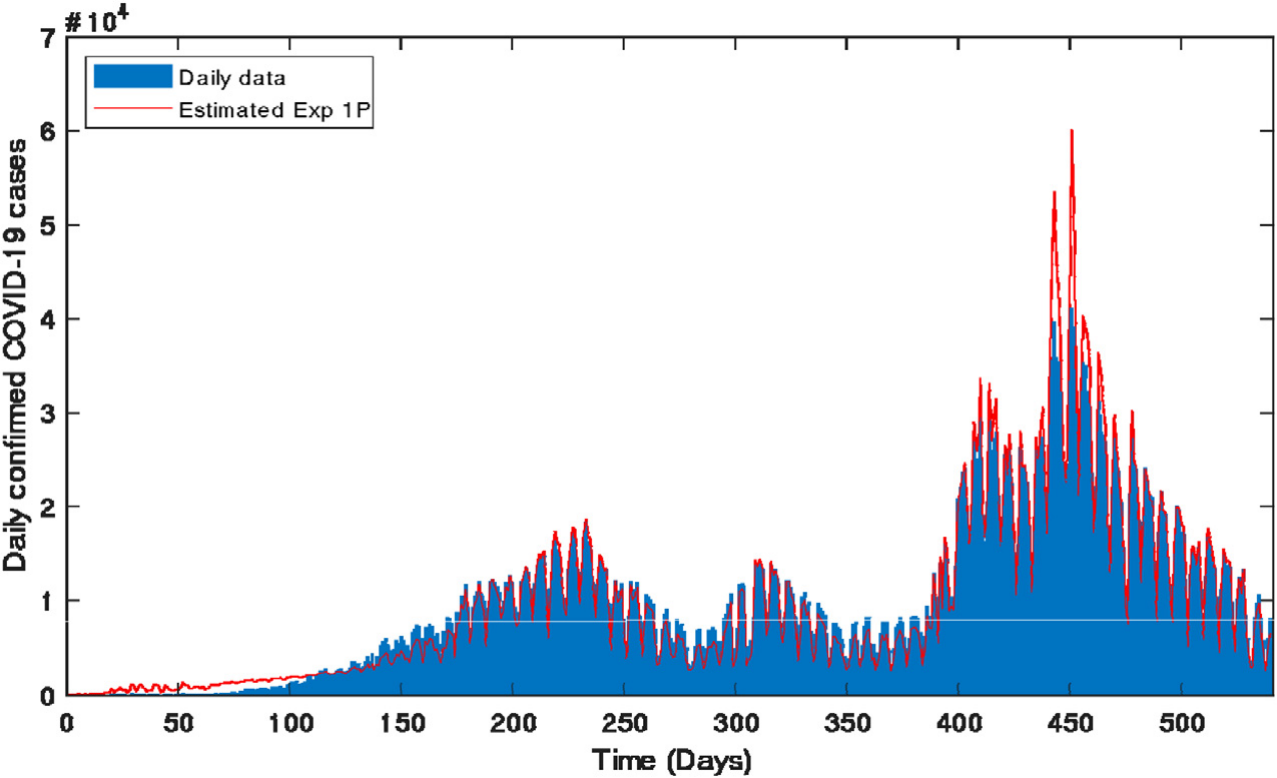
Daily new confirmed cases in Argentina, probabilistic characterization with Exp-1P.

**Fig. 7:**
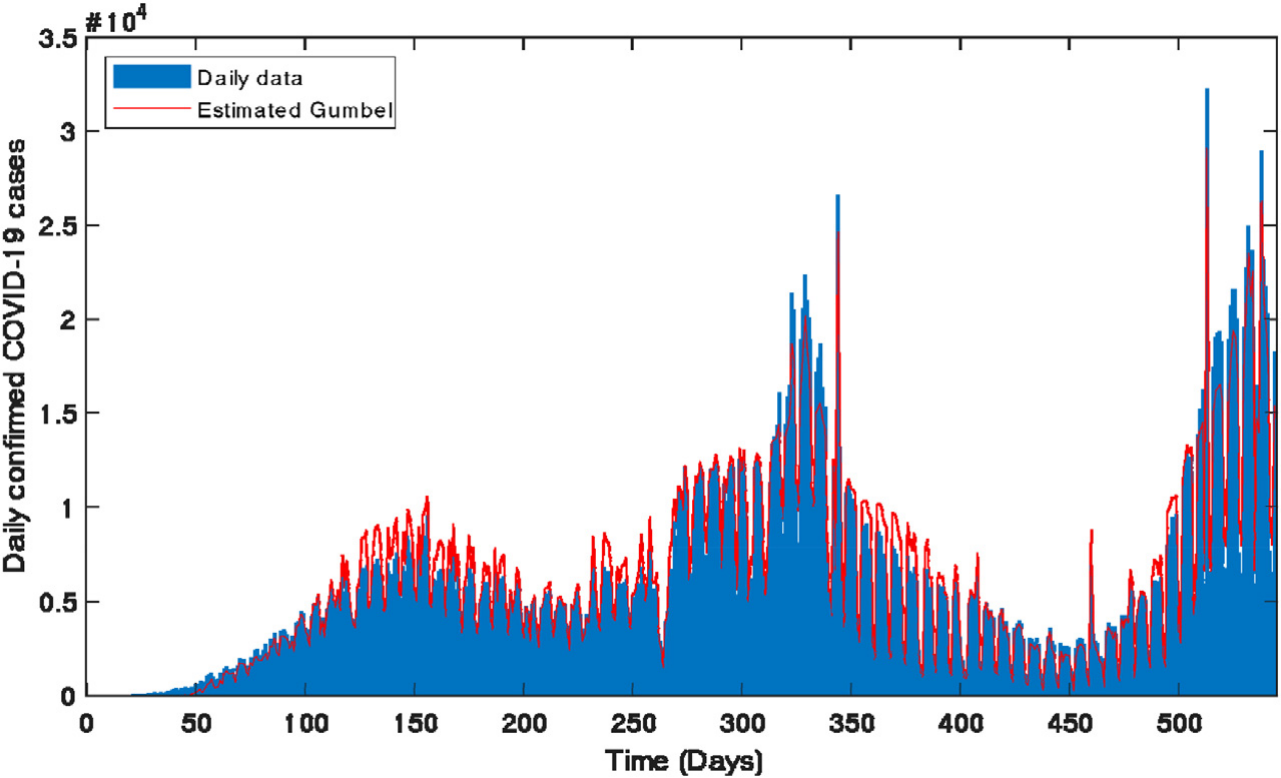
Daily new confirmed cases in Mexico, probabilistic characterization with Gumbel.

Worldwide, COVID-19 coronavirus disease is spreading rapidly in a second and third wave of infections. In this context of increasing infections, it is critical to know the probability of a specific number of cases being reported [3]. Daily data on new confirmed cases of COVID-19 outbreaks in 16 most affected countries: Argentina, Brazil, China, Colombia, Italy, Spain, France [4], Germany, India [5], Indonesia, Iran, Mexico, Poland, Russia, U.K., and the United States from the 20th of January, 2020 to 28th of August 2021 were collected from COVID-19 Dashboard by the Center for Systems Science and Engineering (CSSE) at Johns Hopkins University (JHU) (https://coronavirus.jhu.edu/). A sample of more than ten thousand daily data is utilized to validate the proposed models. Figs. 1 to 4 shows an example of fit frequency analysis. Comparison between fit proposed models Exp-1P, Exp-2P and Gumbel with daily data on confirmed cases of COVID-19 showed in Figs. 5 to 7.

Very specific studies on COVID-19 forecasting are currently available. It is common to use autoregressive models of the type ARMA(p,q). For example [6], utilize Hidden Markov Chain Models of Moroccan data y [7] using Recurrent Neural Networks; these studies are “forecasting" models. However, there are few studies on the probability of a specific number of infections happening in a day. This is one of the highlights of this dataset. It is proposed to use a frequency analysis to assign a probability of occurrence (infection) of a very particular day in a specific country.

A theoretical frequency analysis means to fit a series of data to a probability distribution function P(x), which represents the probability of occurrence of a random variable. This procedure must be applied when it is desirable to know an event associated with a return period greater than the maximum length of data record; this is why it is called theoretical because it is not possible to estimate the event using an empirical frequency table. There are several probability distribution functions. Those most successfully used are: normal, log-normal, exponential, gamma, Pearson type III (or three-parameter gamma), log-Pearson type III and those of extreme values types I, II and III; or Gumbel, Frechet and Weibull, respectively. Mixed probability functions are also used, i.e. they can take into account two or three data sets. For daily covid data we propose to use the extreme distributions shown below.

### 1.1 Gumbel distribution

(1) $$F\left(x\right)=e^{-e^{-\alpha \left(x-\beta \right)}}$$

Where

(2) $$\alpha =\frac{1.2825}{s};\;\beta =\bar{x}-0.45s$$

Where s is the standard deviation and $\bar{x}$ is the mean. α is the scale parameter. β is the shape parameter. Then to equal the probability function of the return period $\;P=1-\frac{1}{T}$ with the distribution function is.

(3) $$F\left(X\right)=\int_{0}^{X}f\left(x\right)\text{dx}=P\left(x\leq X\right)=1-\frac{1}{T}$$

And solving *x*

(4) $$x=\beta -\frac{1}{\alpha }\left[\text{lnln}\frac{T}{T-1}\right]$$

### 1.2 Exponential distribution

(5) $$F\left(x\right)=1-e^{-\beta x}$$

Where

(6) $$\beta =\frac{1}{\bar{x}}$$

$\bar{x}$ is the mean. β is the location parameter. According to the return period is:

(7) $$F\left(X\right)=\int_{0}^{X}f\left(x\right)\text{dx}=P\left(x\leq X\right)=1-\frac{1}{T}$$

And solving x

(8) $$x=\frac{1}{\beta }\text{Ln}\left(\text{Tr}\right)$$

### 1.3 Exponential II distribution

(9) $$F\left(x\right)=1-e^{-\left(\frac{x-\mu }{\beta }\right)}$$

Where

(10) $$\beta =s;\;\mu =\bar{x}-s$$

Where s is the standard deviation and $\bar{x}$ is the mean. β is the scale parameter. μ is the shape parameter. According to the return period is:

(11) $$F\left(X\right)=\int_{0}^{X}f\left(x\right)\text{dx}=P\left(x\leq X\right)=1-\frac{1}{T}$$

And solving x

(12) $$x=\mu +\left(-\text{Ln}\left(\frac{1}{\text{Tr}}\right)\beta \right)$$

## 2 Experimental design, materials and methods

Generalized Extreme-Value Distribution Type-1 (Gumbel) [8] and Exponential models were introduced to analyze the probability of new daily confirmed cases. The data presented in this document for each country provide the daily probability of rate incidence [9]. In addition, the frequencies of historical events expressed as a return period in days of the complete data set is provided. Table 1 shows the estimation of the parameters of the distributions used. This probabilistic analysis comes from the frequency analysis in each of the countries. Only some countries are shown here as examples. The total of the probabilistic analysis can be obtained from the database of this paper. If a series of extreme values is used, the maximum data recorded in each day must be used. This series is used when the design must be based on the most adverse conditions. The empirical return period of this data series is obtained with the following expression proposed by Hosking et al. [10].

(13) T=(n+1)/m

Where

*T* is the empirical return period, in days
*n* is the total number of data in each country
*m* is the order-number in a list from high to low value

**Table 1:** Parameters for fit proposed models Exponential (1, 2 parameters) and Gumbel.

		Gumbel		Exponential 2p		Exponential 1p
Country	Total analyzed data	Scale parameter	Shape parameter	Scale parameter	Shape parameter	Location parameter
Argentina	540	7448.0	4556.5	8284.5	1267.9	0.0001046
Brazil	546	300,052.0	13,869.0	25,216.4	13,326.7	0.0000259
China	581	126.8	460.6	837.5	-674.9	0.0061500
Colombia	537	7106.9	4143.5	9114.9	1581.3	0.0001090
France	579	11,938.3	5691.2	15,305.6	-2732.5	0.0000790
Germany	576	6768.8	2873.0	8771.6	-1593.2	0.0001440
India	573	44,240.5	45,262.6	82,295.6	-25,555.0	0.0000176
Indonesia	541	7890.7	2856.5	10,116.2	-2707.4	0.0001350
Iran	553	6942.9	4595.5	8901.2	-300.1	0.0001160
Italy	573	7049.8	3789.4	9041.7	1183.6	0.0001273
Mexico	544	2498.0	4529.4	5174.9	796.3	1867.00
Poland	539	6089.8	1841.6	7810.4	2454.2	0.0001867
Russia	572	6366.8	8022.4	8165.7	3531.2	0.0000855
Spain	571	9843.1	2891.5	12,619.4	-4049.1	8570.26
Turkey	532	10,352.3	4252.0	13,277.3	-3050.5	0.0000978
U. Kingdom	572	10,913.0	5271.3	13,991.1	-2423.8	0.0000860
United States	580	50,807.4	36,334.4	65,137.7	508.7	65,646.35

When historical records of a phenomenon are used, defined as daily data, they should be assigned a return period according to their observed cumulative frequencies (frequencies table). To calculate it, it is assumed that the frequency or recurrence interval of each observed event, allows assigning a return period to each data. This is known as the observed (empirical) return period. Since the return period has a completely probabilistic definition, in its mathematical form *T* of a daily event *x*, it should be defined as the inverse of the probability P(x) of that event *x* to occur. This means that the probability of being equalized or exceeded by another event *x* must be expressed as:

(14) T=1/P(x)=1/P(X≥x)

## Ethics Statements

The authors paid attention to the ethical rules in the study. There is no violation of ethics. The authors declare that this work does not involve the use of human subjects or experimentation with animals.

## CRediT Author Statement

**Daniel Canton Enriquez** and **Alfonso Gutierrez-Lopez:** Designed the model and the computational framework. All Authors analyzed the data, carried out the implementation and performed the calculations; **Alfonso Gutierrez-Lopez** and **Martin Muñoz Mandujano:** Wrote the manuscript with input from all authors; **Ivan Gonzalez Garcia, Jose A. Niembro-Ceceña1** and **Jorge Arcadia Guerrero:** Were in charge of overall direction and planning.

## Funding

This work was financially supported by Consejo Nacional de Ciencia y Tecnología, CONACYT, Mexico.

## Declaration of Competing Interest

The authors declare that they have no known competing financial interests or personal relationships that could have appeared to influence the work reported in this paper.

## References

[1] Dong E., Du H., Gardner L. (2020). An interactive web-based dashboard to track COVID-19 in real time. Lancet Infect. Dis..

[2] Lynch C.J., Gore R. (2021). Short-range forecasting of COVID-19 during early onset at county, health district, and state geographic levels using seven methods: comparative forecasting study. J. Med. Internet Res..

[3] Perc M., Gorišek Miksić N., Slavinec M., Stožer A. (2020). Forecasting COVID-19. Front. Phys..

[4] Ceylan Z. (2020). Estimation of COVID-19 prevalence in Italy, Spain, and France. Sci. Total Environ..

[5] R. Gupta, S.K. Pal, (2020). Trend Analysis and Forecasting of COVID-19 outbreak in India. MedRxiv. doi:10.1101/2020.03.26.20044511.

[6] Marfak A., Achak D., Azizi A., Nejjari C., Aboudi K., Saad E., Youlyouz-Marfak I. (2020). The hidden Markov chain modelling of the COVID-19 spreading using Moroccan dataset. Data Brief.

[7] Hawas M. (2020). Generated time-series prediction data of COVID-19′ s daily infections in Brazil by using recurrent neural networks. Data Brief.

[8] Molina-Aguilar J.P., Gutierrez-Lopez A., Raynal-Villaseñor J.A., Garcia-Valenzuela L.G. (2019). Optimization of parameters in the generalized extreme-value distribution type 1 for three populations using harmonic search. Atmosphere.

[9] Jalilian A., Mateu J. (2021). A hierarchical spatio-temporal model to analyze relative risk variations of COVID-19: a focus on Spain, Italy and Germany. Stoch. Environ. Res. Risk Assess..

[10] Hosking J.R.M., Wallis J.R., Wood E.F. (1985). Estimation of the generalized extreme-value distribution by the method of probability-weighted moments. Technometrics.

[11] D. Canton, J. Niembro, M. Muñoz, D. Alarcon, J. Arcadia, I. Gonzalez, A. Montes, A. Gutierrez, (2021). Frequency analysis for confirmed cases of COVID-19 in 17 countries. (Version 1.0.0) [Computer software]. doi:10.13140/RG.2.2.14228.63361

